# Critical role of SP thymocyte motility in regulation of thymic output in neonatal *Aire*^−/−^ mice

**DOI:** 10.18632/oncotarget.13909

**Published:** 2016-12-11

**Authors:** Rong Jin, Abudureyimujiang Aili, Yuqing Wang, Jia Wu, Xiuyuan Sun, Yu Zhang, Qing Ge

**Affiliations:** ^1^ Department of Immunology, Key Laboratory of Medical Immunology, Ministry of Health. Peking University Health Science Center, Beijing, China

**Keywords:** Aire deficiency, neonatal thymic emigration, thymocyte motility, CCR7, Immunology and Microbiology Section, Immune response, Immunity

## Abstract

Autoimmune regulator (Aire) is essential in the perinatal period to prevent the multiorgan autoimmunity. Here we show that Aire-regulated single positive thymocyte trafficking in neonatal period is critical for thymic egress. Reduced thymic emigration was found in Aire^−/−^ mice during neonatal period, leading to enhanced homeostatic expansion of peripheral T cells as early as 2 weeks of age. In neonatal Aire^−/−^ mice, thymic expression of CCR7 ligands were dramatically reduced, resulting in decreased thymocyte motility and thymocyte emigration. This reduction of thymic egress in Aire^−/−^ mice was alleviated beyond 3 weeks of age by an early upregulation of S1P_1_ signaling. As the numbers and quality of thymic emigrants are essential for the establishment and maintenance of peripheral tolerance, the reduced thymic emigration during neonatal period may deteriorate autoimmunity caused by the emigration of autoreactive T cells.

## INTRODUCTION

The autoimmune regulator (Aire) is found as a transcription factor that plays an essential role in the regulation of central tolerance and mTEC differentiation [1]. Aire is expressed in medullary thymic epithelial cells (mTECs) and regulates the expression of a significant fraction of tissue-restricted antigen (TRA) [2–5]. Aire deficiency leads to failure in negative selection of single positive (SP) thymocytes and the development of a variety of autoantibodies and lymphocytic infiltration in multiple tissues both in human and in mice [2–3]. Aire is also involved in mTEC differentiation. For instance, *Aire*^−/−^ mice lack terminally differentiated mTECs and have an aberrant expression of a number of chemokines in mTECs, including CCR4 and CCR7 ligands [2, 6–7]. A developmental blockage in CD4 SP thymocytes was found in Aire-deficient mice. *Aire*^−/−^ thymus also showed a delay in CD4^+^ T cell emigration as early as 5-day after birth [6]. As Diane Mathis's group showed that Aire was essential in perinatal period to prevent the multiorgan autoimmunity [8], the defect in T cell egress during this period implicates that SP thymocyte emigration may also have a role in preventing autoimmunity.

To further clarify the signals that regulate neonatal thymic emigration and to investigate whether Aire deficiency affects thymic egress of CD4 SP thymocytes in adult mice, we used FITC intrathymic injection and RAG2p-GFP transgenic mouse model to monitor the numbers of recent thymic emigrants (RTEs) at various ages of mice. We showed that in Aire-deficient mice, reduced thymocyte emigration only occurred within the first 3 weeks after birth. This reduced thymic egress in neonatal *Aire*^−/−^ mice was likely associated with impaired SP thymocyte motility caused by decreased thymic expression of CCR7 ligand. An early upregulation of sphingosine-1-phosphate receptor 1 (S1P_1_) expression in *Aire*^−/−^ thymocytes alleviated the egress defect. Thus, CCR7 and S1P_1_ signals were both critical for thymic emigration during mouse ontogeny.

## RESULTS

### Neonatal *Aire*^−/−^ mice had reduced numbers of RTEs

**Figure 1:**
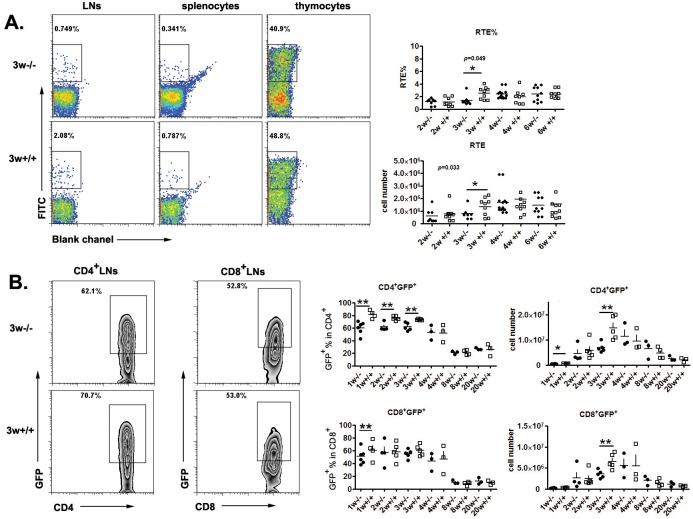
Thymic output decreased in neonatal *Aire*^**−/−**^ mice **A.** FITC was injected into the thymus of *Aire*^−/−^ mice and littermates, twenty-four hours later, FITC^+^ cells in the thymus and periphery were collected to calculate the daily emigration rate(RTE%) and newly migrated cell number .Representative dot plots of FITC^+^ cells from 3-week old mice are shown on the left and the ratio and cell numbers of RTEs on the right. 8-10 pairs of littermates were used for each time point. **B.** The Aire^−/−^RAG-GFP mice were used to detect the recent thymic emigrants during mouse ontogeny. Representative zebra plots of GFP^+^ cells in the lymph nodes from 3-week old mice are shown on the left The ratio and absolute number of GFP^+^ cells in CD4^+^ and CD8^+^ T cells from lymph nodes were shown on the right. 3-5 pairs of littermates were detected for each time point. * *p* < 0.05, ** *p* < 0.01.

To investigate thymic egress in *Aire*^−/−^ mice, we used two approaches to measure the numbers of RTEs. The first is FITC intrathymic injection followed by peripheral FITC^+^ cell detection 24 hours later. Compared to WT mice, a significantly smaller daily export rate and RTE (FITC^+^) cell numbers were found in the lymph nodes and spleen of *Aire*^−/−^ mice at 3 weeks of age but not at later time points (Figure 1A). As this method requires complicated surgery and cannot be easily performed in newborn mice, we used RAG2p-GFP transgenic (tg) mice as the second approach to study RTEs. The GFP expression in this line of tg mice is driven by RAG2 promoter. After thymic egress, the time frame of GFP expression in T cells is about 2-3 weeks, enabling an easy tracking of RTEs in mice without manipulation [9–10] We thus crossed RAG2p-GFP tg with *Aire*^−/−^ mice. As shown in Figure 1B, significantly less GFP^+^CD4^+^ RTEs (both percentage and cell number) were found in KO mice than in littermates within 3 weeks after birth. In mice at 4 weeks of age or older, this difference in the number of CD4^+^ RTEs disappeared. The disparities in the cell number and percentage of CD8^+^ RTEs between neonatal *Aire*^−/−^ and wild type (WT) mice were smaller than CD4^+^ RTEs (Figure 1B). The results from FITC (detection of 24-hour-old RTEs) and GFP tg (detection of 1-to-14-day-old RTEs) suggest that only neonatal but not adult *Aire*^−/−^ mice had reduced capacity of thymic emigration of CD4^+^ T cells.

### RTEs of neonatal *Aire*^−/−^ mice were phenotypically and functionally different from WT mice

**Figure 2:**
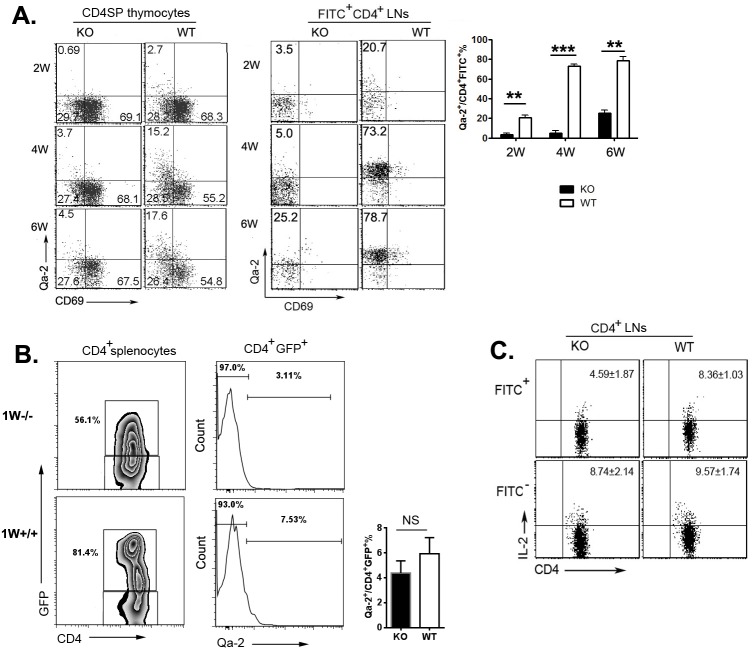
Phenotype alterations of TCRβ^**+**^CD4SP thymocytes and RTEs **A.** CD8-depleted thymocytes from various time points during *Aire*^−/−^ mice and littermates ontogeny were stained anti-CD4, anti-TCR-β, anti-CD69 and anti-Qa-2. The expression of CD69 *versus* Qa-2 was analyzed in TCRβ^+^CD4SP medullary thymocytes(left). Twenty-four hours after FITC injection, CD4^+^FITC^+^ cells in lymph nodes were collected for Qa-2 and CD69 analysis (middle).The ratio of Qa-2 expression on RTEs was shown on the right panel. Data from 5 mice are shown as mean± SD. **B.** Representative zebra plots of GFP expression on CD4^+^ splenocytes from 1-week old *Aire*^−/−^ RAG-GFP mice and littermates was shown on the left panel. Qa-2 expression on CD4^+^GFP^+^RTEs were shown in the middle. The ratio Qa-2^+^ in CD4^+^ GFP^+^ cells were summarized on the right panel. Data from 5 mice are shown as mean ± SD.**C.** 3-week old *Aire*^−/−^ mice and littermates were intrathymic injected FITC. Twenty-four hours later, CD4^+^ cells in lymph nodes were collected and stimulated with PMA and ionomycin for 4 hours. After intracellular staining of IL-2, CD4^+^FITC^+^ and CD4^+^FITC^−^ cells were gated for IL-2 analysis. Data from 5 mice are shown as mean±SD. ** *p* < 0.01, *** *p* < 0.001. *NS*, no significance.

We have previously demonstrated that CD4^+^ SP thymocytes could be resolved into four subsets, SP1 (6C10^+^CD69^+^), SP2 (6C10^−^CD69^+^), SP3 (CD69^−^Qa-2^−^), and SP4 (CD69^−^Qa-2^+^), with a gradual increase in the capabilities of proliferation, cytokine production, survival, and egress [11]. As thymocytes at SP4 stage expressed highest level of S1P_1_ and CD62L and have been shown to be the main population leaving the thymus [12], a severe developmental blockage at the transition from SP3 to SP4 in adult *Aire*^−/−^ mice may implicate that reduced thymic egress in these mice is due to the absence of mature SP4 thymocytes in the thymus. To examine this possibility, we first determined the percentages of Qa-2^+^CD69^−^ (SP4) cells in *Aire*^−/−^ and wild type (WT) thymus at 2, 4, and 6 weeks after birth. As shown in Figure 2A, about 2.7% of CD4^+^ SP thymocytes in 2-week old WT mice were Qa-2^+^. This percentage was increased to more than 15% in WT thymus at 4- and 6-week of age (Figure 2A). However, the ratio of Qa-2^+^CD4^+^ SP thymocytes (SP4) in *Aire*^−/−^ mice was 4-5-fold less than that in WT mice at all time points examined (Figure 2A). Similar percentages of SP1, SP2, and SP3 were found between WT and KO mice. As comparable *in situ* apoptosis was found in SP3/4 thymocytes in *Aire*^−/−^ and WT mice (data not shown), this result in young mice further supports the SP3-to-SP4 developmental blockage in *Aire*^−/−^ thymus.

At 1-week of age, the majority of RTEs from WT as well as *Aire*^−/−^ mice bear a Qa-2^−^CD69^−^ phenotype (Figure 2B). As the mice grew older, the percentage of WT Qa-2^+^ thymic emigrants was significantly elevated (from 20.7% at 2-week to 78.7% at 6-week old) with the increase of Qa-2^+^ SP cells in the thymus (Figure 2A). The percentage of *Aire*^−/−^ FITC^+^Qa-2^+^CD4^+^ or GFP^+^Qa-2^+^CD4^+^ thymic emigrants, however, did not dramatically increase, neither was *Aire*^−/−^ Qa-2^+^ SP cell ratio in the thymus (Figure 2A and data not shown). At 6-week of age, more than 70% of RTEs in *Aire*^−/−^ mice were remained Qa-2^−^. In addition, *Aire*^−/−^ RTEs produced lower level of IL-2 than WT RTEs (Figure 2C). Both WT and mutant mice had similar number of total thymocytes and similar expressions of CD25, CD69, and CD44 in RTEs (Figure S1A-S1C). These data suggest that the SP3-to-SP4 blockage in *Aire*^−/−^ mice may result in thymic emigration of cells with phenotypic and functional immaturity. But lacking Qa-2 expression in *Aire*^−/−^ RTEs may not be the main reason for reduced thymic egress in these mice.

### Reduced thymic output in neonatal *Aire*^−/−^ mice results in homeostatic proliferation of peripheral T cells

**Figure 3:**
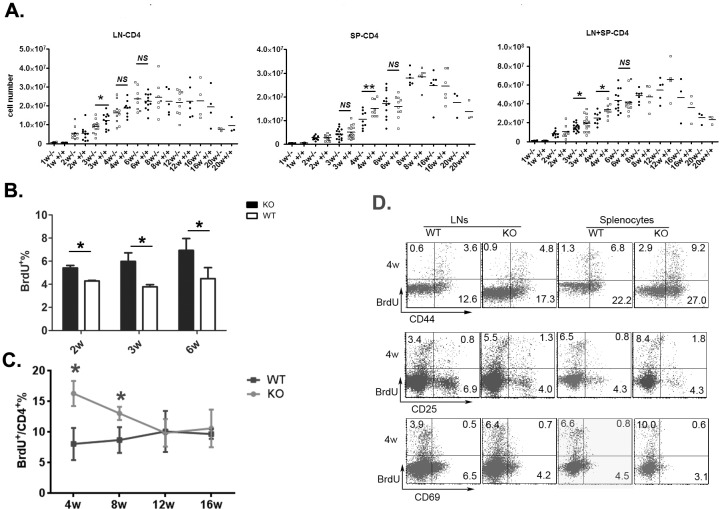
The homeostasis proliferation of peripheral CD4^**+**^ cells in Aire KO mice **A.** The kinetics of CD4^+^ cells numbers during mouse ontogeny. The absolute number of CD4^+^ T cells in lymph nodes (mesenteric, inguinal, and axillary nodes)(left) and spleen(middle) or the total number of CD4^+^ T cells in lymph nodes and spleen(right) were shown from 1-20 weeks old *Aire*^−/−^ mice and littermates. 3-10 pairs of littermates were detected for each time point. **B.** Mice received two intraperitoneal injections of BrdU (1 mg each at 4-h intervals). Lymph nodes were harvested 1 h after the second injection. BrdU^+^ cells in CD4^+^ T cells were shown. Data from 5 mice are shown as mean±SD. **C.**-**D.** Mice were given BrdU at 1 mg/ml in drinking water for 7 days. The turnover rate(C) and the expression of CD44,CD25 and CD69(D) in peripheral CD4^+^ T cells from 4-week old *Aire*^−/−^ mice and littermates were shown. Data are presented as mean±SD * *p* < 0.05, *NS*, no significance.

We next determined whether reduced CD4^+^ RTEs in *Aire*^−/−^ mice resulted in decreased total T cell numbers in the periphery. In WT mice, the number of CD4^+^ T cells in the lymph nodes and spleen gradually increased until the mice reached 8 weeks of age (Figure 3A). In *Aire*^−/−^ mice, this T cell number elevation was significantly delayed at 3 and 4 weeks of age. But at 6 weeks of age or older, both mice showed similar number of peripheral CD4^+^ T cells (Figure 3A). To examine whether reduced thymic output and reduced total CD4^+^ T cells during neonatal period lead to enhanced homeostatic proliferation of peripheral T cells, BrdU was injected into mice 1 and 5 hours before analysis. As shown in Figure 3B, the percentage of BrdU^+^ peripheral T cells was significantly higher in *Aire*^−/−^ than in WT mice at 2-, 3-, and 6-week of age. We also measured T cell turnover rate by treating the mice with BrdU-containing water for 7 days. Compared to WT mice, T cell turnover was enhanced in 4- and 8-week old *Aire*^−/−^ mice but reached similar level at 12- and 16-week of age (Figure 3C). In addition, the majority of BrdU^+^ cells were CD44^hi^CD69^−^CD25^−^ T cells (Figure 3D). These results suggest that reduced thymic egress is associated with enhanced lymphopenia-driven peripheral T cell proliferation in *Aire*^−/−^ mice.

### The altered expression of CCR4 and CCR7 ligands in *Aire*^−/−^ mice contributes to the defects in early thymic egress

**Figure 4:**
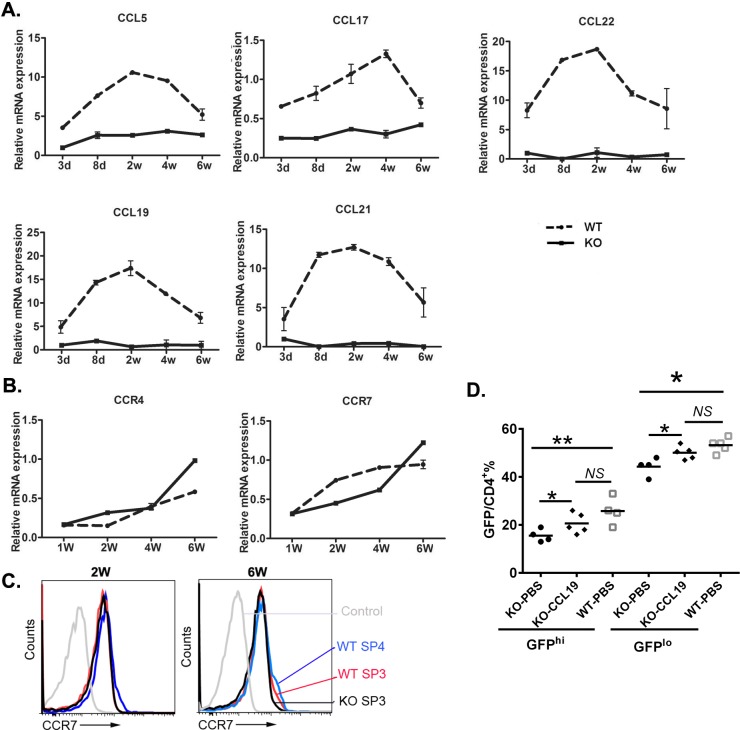
The chemokines and receptors expression in the thymus during mouse ontogeny **A.** The mRNA expression level of the CCR4 ligands CCL5, CCL17, and CCL22 and the CCR7 ligands CCL19 and CCL21 in whole thymi from *Aire*^−/−^ mice and littermates were analyzed by qPCR. K-8 was used as internal standard. **B.** The mRNA expression of CCR4 and CCR7 in whole thymi. GAPDH was used as internal standard. The experiment was repeated for 4 times and a representative result is shown. **C.**The cell surface CCR7 expression in SP3 and SP4 thymocytes from 2- or 6-week old *Aire*^−/−^ and littermate were shown in overlay histograms. **D.** 1μg CCL19 or PBS was intrathymically injected to the 2 weeks old *Aire*^−/−^ RAG2p-GFP mice,littermate *Aire*^+/+^ RAG2p-GFP mice were administrated with PBS. 3 days later, CD4^+^ T cells were collected from the lymph nodes, and GFP fluorescence intensity was used to determine GFP^hi^ and GFP^lo^ RTEs cells. *NS*, no significance. * *p* < 0.05, ** *p* < 0.01.

CCR4 and its ligands CCL5, CCL17, and CCL22; CCR7 and its ligands CCL19 and CCL21 are known to be involved in thymocyte trafficking to or within the medulla while CCR7 is also required for thymic egress in neonatal but not adult mice [13]. As mTECs in adult *Aire*^−/−^ mice were reported to have defects in producing these ligands [6, 14], we investigated the expression profile of these chemokines in neonatal mice. In WT thymus, the expressions of CCL5, CCL22, CCL19, and CCL21 quickly increased during the first week after birth and reached their peak values on day 8 or 14. The upregulation of CCL17 was slightly slower and the expression peaked around week 4. In contrast, *Aire*^−/−^ thymus revealed significantly lower levels of all chemokines examined during the entire 6-week study period (Figure 4A). No upregulation of these chemokines was observed. The thymic expression of CCR4 and CCR7 was gradually increased as the mice reached 6-week of age. However, WT and *Aire*^−/−^ thymus did not show any difference in the expression of these two chemokine receptors (Figure 4B). In particular, the surface expression of CCR7 in *Aire*^−/−^ SP3 thymocytes was comparable to WT SP3 and even SP4 thymocytes at both 2- and 6-week of age (Figure 4C).

To investigate whether these reduced chemokines in *Aire*^−/−^ thymus contribute to the defects in neonatal thymic egress, CCL19 was administered into 2-week-old *Aire*^−/−^ RAG2p-GFP mice *via* intrathymic injection. As shown in Figure 4D, the differences in the ratio of peripheral GFP^+^ RTEs between *Aire*^−/−^ and WT mice were diminished in the *Aire*^−/−^ groups with CCL19 administration. These data suggest that the defect in the production of CCR7 ligands by *Aire*^−/−^ thymic epithelial cells, but not SP thymocytes, contributes to the reduced thymic output during neonatal period.

### SP thymocytes motility was reduced in neonatal *Aire*^−/−^ mice

**Figure 5:**
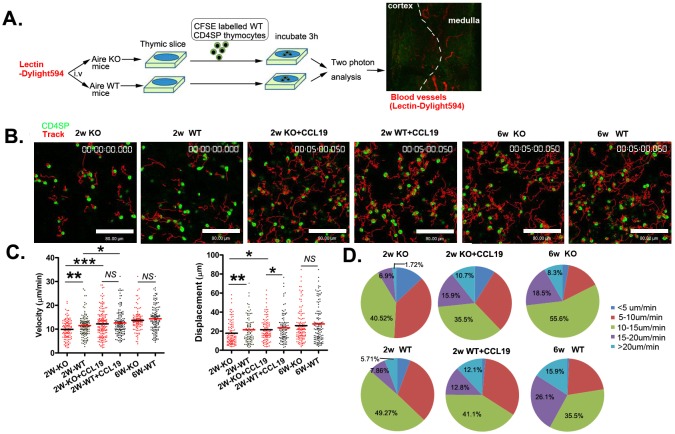
The reduced motility of CD4SP thymocytes within the thymic tissue of neonatal *Aire* KO mice **A.** The experimental design used to analyse CD4SP thymocyte migration in sliced thymic tissues using a two-photon microscope. Dylight594 conjugated lectin was injected to the *Aire*^−/−^ and *Aire*^+/+^ mice just prior to sacrifice. 4×10^5^ CFSE-labelled CD4SP cells were loaded onto thymic slices from Aire^−/−^ or Aire^+/+^ mice, and the cells on the slice were incubated for 3h at 37°C/5% CO_2_ to allow cells to enter the tissue. For CCL19 rescue assay, CD4SP cells were incubated with 200ng/ml CCL19 and then loaded on the thymic tissues. The slice was imaged by two photon microscope. The thymic structures were visualized by the labelled blood vessels. **B.** The movements of individual cells were tracked. CD4SP (green). Tracks (red). Scale bar, 80 μm. **C.** The average velocity of CD4SP in the medulla are shown in left panel. Bars indicate the median. Average displacements from the origin plotted are also shown in right panel. The raw tracking data for CD4SP thymocytes are from three independent experiments. (2 weeks-old *Aire*^−/−^, *n* = 116, *Aire*^+/+^ mice, *n* = 140; 2 weeks-old with CCL19 *Aire*^−/−^, *n* = 168 and *Aire*^+/+^ mice,*n* = 140, 6 weeks-old *Aire*^−/−^,*n* = 108 and *Aire*^+/+^ mice, *n* = 138). **D.** Profiles of the migration velocities of CD4SP cells on different thymic slices. *p* values were calculated by Mann-Whitney U-test, * *p* < 0.05, ** *p* < 0.01, *** *p* < 0.001. *NS*, no significance.

Up to now, it is not well understood how CCR7 and its ligands drive SP thymocyte emigration as the ligands are highly expressed in thymic medulla. Recently, it was found that in addition to guide the trafficking of T cells, the binding of CCR7 with its ligands also stimulate the basal motility of T cells in the lymph nodes, facilitating T cell scanning of dendritic cells for cognate antigen [15–18]. Whether this chemokinetic function also contributes to thymic egress is not known. We thus purified CD4 SP thymocytes from wild type mice and seeded them on thymic slices derived from *Aire* KO and WT mice. Three hours later, the slices were monitored by two photon microscopy. The mice just prior to sacrifice were injected with fluorescence-labeled lectin to label the blood vessels. The medulla can be readily distinguished from the cortex, based on the size and orientation of blood vessels at the cortical-medullary junction (CMJ) (Figure 5A). The tracks of CD4 SP thymocytes in thymic slice were shown in Figure 5B. The average cell velocities of CD4 SP thymocytes were 11.4 μm min^−1^ and 14.3 μm min^−1^ in slices obtained from *Aire^+/+^* mice at 2- and 6-week of age respectively (Figure 5C, Supplementary Movies 2,4). Compared to SP cell movement in WT thymic slice, those in *Aire^−/−^* thymic slice (2-week of age) was significantly slower (9.6 μm min^−1^ in KO slice *vs* 11.4 μm min^−1^ in WT slice). Similar reduction in the average displacement of SP thymocytes was found in *Aire^−/−^* thymic slice at 2-week of age (17.8 μm in KO slice *vs* 21.4 μm in WT slice) (Figure 5C, Supplementary Movies 1, 2). Interestingly, the basal T cell motility was comparable in WT and KO thymic slices obtained from 6 weeks old mice (Figure 5C, Supplementary Movies 3, 4), being consistent with reduced thymic egress in *Aire*^−/−^ mice at neonatal period but not adulthood.

As CCL19 intrathymic administration rescued the thymic egress in 2-week old *Aire^−/−^* mice, we investigated whether CCL19 could promote the basal motility of CD4SP thymocytes in thymic slices. WT CD4 SP thymocytes were pretreated with CCL19 before being added into the thymic slices obtained from 2-week old of *Aire* KO and WT mice (Supplementary Movies 5, 6). The average cell velocity in both slices was elevated and reached comparable levels (*Aire^−/−^*: 9.6 μm min^−1^ without CCL19 *vs*. 12.2 μm min^−1^ with CCL19; *Aire^+/+^*: 11.46 μm min^−1^ without CCL19 *vs* 12.59 μm min^−1^ with CCL19) (Figure 5C). Similar results were obtained when analyzing cell displacement (Figure 5C). Furthermore, we found that the proportion of cells with velocity of more than 20 μm min^−1^ was significantly higher in WT than in KO slices obtained from 2-week old mice (WT 5.71% *vs*. KO 1.72%). The pretreatment with CCL19 increased the percentage of cells with a velocity of more than 15 μm min^−1^ in both WT and KO thymic slices (8.62% without CCL19 *vs* 26.6% with CCL19 in KO slice and 13.57% *vs* 24.9% in WT slice) (Figure 5D). In short, our data suggest that altered levels of CCR7 ligands in Aire-deficient mice contribute to reduced basal cell motility, which may ultimately lead to reduced thymic emigration at neonatal period.

### S1P_1_ may contribute to overcome the defect of perinatal thymic egress in *Aire*^−/−^ mice

**Figure 6:**
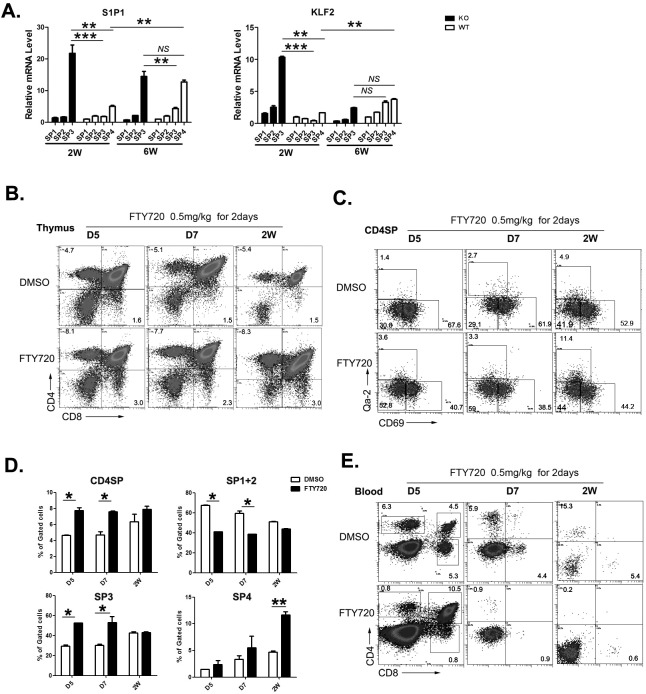
The role of S1P1 signal in neonatal thymic output **A.** The four subgroups of CD4SP thymocytes from 2 and 6-week old *Aire*^−/−^ mice and littermates were sorted. The mRNA expression levels of S1P1 and KLF2 were analyzed by qPCR. GAPDH was used as internal standard. **B.**-**E.** FTY720 (0.5mg/kg) was administrated to wild type mice from D5 to 2 weeks old by intraperitoneally injection for 2 days. **B.**-**D.** 24 hours later, thymocytes were collected for CD4,CD8,CD69 and Qa-2 staining. The ratio of CD4SP in total thymocytes and SP1 to SP4 cells in CD4SP were indicated as mean± SD. * *p* < 0.05. ** *p* < 0.01. **E.** Blood depleted red cells was collected for CD4 and CD8 staining. Representative dot plots were shown. N = 5. * *p* < 0.05, ** *p* < 0.01, *** *p* < 0.001. *NS*, no significance.

Egress from adult thymus is critically dependent on the binding of S1P to its receptor S1P_1_ expressed on SP thymocytes [19–21]. The positioning of mature SP thymocytes near exit site and the egress of these cells from the thymus is driven by dynamin-2-dependent S1P_1_ endocytosis (resensitization) in response to small S1P gradients at corticomedullary junctions [22–23]. The expression of S1P_1_ during ontogeny has not been carefully examined. Whether S1P/S1P_1_ axis plays an important role in neonatal thymic egress is also not clear. As S1P_1_ was significantly upregulated during the transition of SP3 to SP4 thymocytes in adult WT mice, the lack of SP4 thymocytes in *Aire*^−/−^ mice may indicate a reduced level of S1P_1_ expression, which leads to defects in thymic egress. We thus analyzed S1P_1_ expression in WT and *Aire*^−/−^ CD4^+^ SP thymoyctes. In WT mice, the expression of S1P_1_ was low at 2-week and high at 6-week after birth. The majority of S1P_1_ positive cells were Qa-2^+^ SP4 thymocytes, even though more than 70% of thymic emigrants were Qa-2^−^ at 2-week of age (Figure 6A). This implicates that S1P/S1P_1_ axis in neonatal thymic egress may be not as important as that in adult thymic egress. To our surprise, Qa-2^−^ SP3 thymocytes in *Aire*^−/−^ mice expressed significantly higher level of S1P_1_ than in WT mice. The level of S1P_1_ in Aire^−/−^ Qa-2^−^ SP3 cells was even higher than that in WT Qa-2^+^ SP4 cells at 2-week of age. But at 6-week of age, comparable levels of S1P_1_ was found between Aire^−/−^ SP3 thymocytes and WT SP4 thymocytes (Figure 6A). The transcription of S1P_1_ is regulated by Kruppel-like transcription factor (KLF)2. The expression patterns of KLF2 were similar to those of S1P_1_ in WT and KO SP thymocytes, except that at 6-week of age, KLF2 level in Aire^−/−^ SP3 was comparable to WT SP3 and SP4 thymocytes (Figure 6A). As the majority of thymic emigrants in Aire^−/−^ mice were Qa-2^−^ cells, these data suggest that the pre-mature upregulation of KLF2 and S1P_1_ expression in *Aire*^−/−^ SP3 thymocytes may partially overcome the neonatal T cell egress defect resulted from reduced thymic expression of CCR7 ligands.

To further investigate whether S1P/S1P_1_ axis plays an important role in regulating thymic egress during neonatal period, FTY720, an S1P_1_ agonist used in blocking thymic egress in adult mice, was intraperitoneally injected into neonatal WT mice. As shown in Figure 6B, the mice injected with FTY720 revealed an increase in CD4 and CD8 SP thymocytes at 5, 7 days, and 2 weeks after birth. Within CD4 SP subsets, FTY720 treated group showed an increase in SP3 thymocytes at 5 and 7 days while an increase in SP4 thymocytes at 14 days after birth (Figure 6C-6D). In the circulation, both CD4 and CD8 double positive and SP T cells could be seen at 5 days after birth while only SP T cells were seen at 7 and 14 days after birth (Figure 6E). The treatment with FTY720 resulted in about 7-fold decrease in circulating CD4 and CD8 SP T cells, while the peripheral double positive T cells at day 5 were not affected. These results suggest that S1P_1_ signal plays an important role in neonatal thymic egress of SP cells but not DP cells. It further suggests that the enhanced early expression of S1P_1_ in CD4 SP thymocytes may alleviate the egress defect in neonatal Aire^−/−^ mice.

## DISCUSSION

Thymic medulla is a critical region for thymocyte maturation and migration. A single transcription factor, Aire, not only mediates the expression of TRA in mTECs, but also affects the terminal differentiation of mTECs and their expression of functional molecules. Our results demonstrate that the deficiency in Aire results in impaired thymic egress of young murine T cells only during neonatal period. The reduced expression of CCR7 ligand and the resulted defects in basal SP thymocyte motility likely lead to the reduced T cell emigration in Aire^−/−^ mice within 3 weeks after birth. This reduction of thymic egress was then alleviated by an early upregulation of S1P_1_ signaling beyond 3 weeks of age.

Neonatal and adult thymi are different. During neonatal period, the volume of the thymus increases rapidly relative to body size, peaking around the first year of human life and the first 1 to 2 weeks of murine life [24–25]. The most mature Qa-2^+^ CD4 or CD8 SP thymocytes were observed at 1 week old, while they reached the adult level at the age of 4-6 weeks [26]. Thus, the majority of thymic emigrants bear a Qa-2^−^ phenotype during the first two-week after birth.

Besides the differences in neonatal and adult thymic volume and the phenotype of thymic emigrants, the regulation of thymic egress is also different. Egress from adult thymus is critically dependent on the binding of S1P to S1P_1_ that is expressed on mature Qa-2^+^ SP thymocytes [19–21]. Dynamin-2-dependent S1P1 endocytosis (resensitization) and GPCR kinase 2 (GRK2)-mediated receptor phosphorylation and β-arrestins recruitment regulate the positioning and egress of adult S1P_1_^+^ SP thymocytes [22–23]. Thus, in *Aire*^−/−^ thymi beyond 2-3 weeks of age, although the expression of CCR7 ligands remains low, the thymic egress was not different to WT ones as comparable expressions of S1P_1_ were found.

However, signals control neonatal thymic output are less well studied and the role of S1P/S1P_1_ in neonatal mice is unclear. In CCL19-neutralized mice and CCR7-deficient mice, neonatal appearance of circulating T cells was defective [27]. *In vivo* blocking of CXCR4 in newborn mice led to accumulation in the thymus of CD4 SP and fewer CD4 T cells in the spleen [28]. For the export of adult thymocytes, there appears to be no obligatory requirement of CCR7 and CXCR4 [13]. In addition, inhibition of S1P_1_ in adult CCR7- or CCR7L-deficient mice results in the accumulation of mature thymocytes in the cortex. It indicates that CCR7 and CXCR4 are required for thymic egress during neonatal period while S1P_1_ is more critical in adult thymic output [29].

Our data partially support the role of S1P_1_ in adult mice as the expression of this molecule in neonatal SP thymocytes was significantly lower than that in adult ones. However, the application of S1P agonist FTY720 blocked SP thymocyte emigration as early as 5 days after birth, suggesting that during neonatal period, the S1P/S1P_1_ axis already starts to drive young T cell emigration. However, the low expression of S1P_1_ in neonatal T cells may not meet the big demand of T cell export to quickly fill the empty peripheral compartment. It may thus require other chemokines/chemokine receptors (e.g. CCL19/21 and CCR7) to facilitate T cell emigration from the thymus.

SP thymocytes in neonatal *Aire*^−/−^ mice revealed comparable CCR7 when compared to WT mice, but the reduced levels of ligands in the thymus still hampered T cell egress. When neonatal *Aire*^−/−^ mice were administrated with CCL19 *via* intrathymic injection, the defect in thymic output could be alleviated. We further showed that the interaction of CCR7 and its ligands enhanced the basal motility of neonatal SP thymocytes which may subsequently facilitate the emigration of T cells from the thymus. It supports the role of CCR7/CCL19 axis on thymic egress during neonatal period. It further indicates that CCR7-driven cell motility contributes significantly to T cell emigration.

Furthermore, the numbers and quality of thymic emigrants are essential for the establishment and maintenance of peripheral tolerance. In particular, T cell tolerance induction in skin appears early before 15 days after birth as extensive migration of thymic emigrants to peripheral tissues only occurs during this period [30]. Thus, in addition to impaired central tolerance, the reduced thymic egress and the resulted increase of lymphopenia-driven T cell proliferation in neonatal *Aire*^−/−^ mice likely affects the establishment of peripheral tolerance in these mice.

Taken together, CCR7/ligands and S1P/S1P_1_ may work in concert to promote thymic egress during neonatal period while the latter become the main driving force in T cell emigration in adult mice.

## MATERIALS AND METHODS

### Mice

C57BL/6 mice were purchased from Peking University Health Science Center and Vital River Lab Animal Technology Company (Beijing, China). *Aire^tm1.1Doi^*^−/+^ and FVB-Tg (Rag2-EGFP) 1Mnz/J mice were purchased from Jackson Laboratory (Bar Harbor, ME). FVB-Tg (Rag2-EGFP) 1Mnz/J mice were backcrossed 10 generations to the C57BL/6 background (termed as RAG2p-GFP in this paper). Aire^−/+^ mice were bred with C57BL/6 to generate *Aire*^−/−^ and *Aire*^+/+^ littermate mice. Aire^−/+^ mice were bred with Rag2p-GFP to generate *Aire*^−/−^ Rag-GFP and *Aire*^+/+^ Rag-GFP littermate mice. The animals were kept in a specific pathogen-free facility at Peking University Health Science Center (Beijing, China). All the animal procedures were conformed to the Chinese Council on Animal Care Guidelines and the study was approved by the ethics committee of Peking University Health Science Center with an approval number of LA2014178.

### Reagents

Alexa Fluor@ 647-conjugated anti-mouse Qa-2 was purchased from BioLegend (San Diego, CA). Anti-Qa-2-FITC(H1-1-2),CD25-PE(PC61),CCL19-Ig, biotinylated goat anti-human Ig were purchased from eBioscience, IL-2-percp (JES6-5H4) was from BioLegend. Annexin V and PI were purchased from Biosea (Beijing, China). All other antibodies used in the study were purchased from BD PharMingen (San Diego, CA).

### RTE analysis

Two methods were used to characterize RTEs. 1) Intrathymic injection of FITC. 2-6 weeks old Aire^−/−^ and littermate Aire^+/+^ mice were anesthetized *via* intraperitoneal injection. An incision was made in the sternum to reveal the thymus, and 10 μl of FITC (1 mg/ml in PBS) was injected into each thymic lobe with a 30-gauge needle. The skin was closed with surgical glue. Twenty-four hours later, the mice were sacrificed and their thymus, spleen and lymph nodes were removed. FITC^+^CD4^+^ T cells in the lymph nodes and spleen were collected for phenotype analysis. Only animals with more than 20% of FITC^+^ thymocytes (typically 30-70%) were included in experimental groups. RTE numbers were calculated as: FITC^+^ splenocytes plus twice FITC^+^ lymph node cells (pooled from mesenteric, inguinal, and axillary nodes). The emigration rate of RTE = measured peripheral RTE / FITC^+^ thymocytes [31]. 2) Aire^−/−^RAG2p-GFP transgenic mice. For the thymic emigration analysis, the GFP fluorescence intensity was used to determine GFP^+^ (RTEs) and GFP^−^ (non-RTEs) cells during 1 to 20 weeks ontogeny. For the chemokine rescue assay, 10 μl of CCL19 (50μg/ml in PBS) was intrathymicly injected into each thymic lobe of 2 weeks old Aire^−/−^ RAG2p-GFP and littermate Aire^+/+^ RAG2p-GFP mice. 3 days later, CD4^+^ T cells were collected from the lymph nodes, and GFP fluorescence intensity was used to determine GFP^hi^ and GFP^lo^ RTEs cells.

### *In vivo* BrdU labeling

For continuous BrdU administration, mice were given BrdU (Sigma Chemicals Co.) at 1 mg/ml in drinking water for 7 days. The BrdU-containing water was protected from light and changed daily [32]. For short term BrdU administration, mice received two intraperitoneal injections of BrdU (1 mg each at 4-h intervals). Lymph nodes were harvested 1 h after the second injection. BrdU incorporation was detected using the APC BrdU Flow Kit (BD Pharmingen). After surface staining, cells were fixed and permeabilized with Cytofix/Cytoperm buffer for 15-30 min on ice and then treated with DNase to expose incorporated BrdU. Subsequently, cells were stained with APC-conjugated anti-BrdU Ab.

### Chemokines and receptors expression in whole tissues by real-time quantitative PCR (qPCR)

Thymi were dissected from 3-day-old to 6-wk-old wildtype (WT) and Aire KO mice. RNA was isolated using TRIzol (Invitrogen/Life Technologies) and reverse-transcribed to cDNA using the SuperScript III Reverse Transcriptase (Invitrogen/Life Technologies). qPCR was performed with the Applied Biosystems Prism 7900 SDS instrument using a qPCR SYBR Green Core Kit (Eurogentec) according to the manufacturer's instructions. Primers used to amplify K8, CCL5, CCL17, CCL22, CCL19, CCL21, CCR4, CCR7, KLF2, S1P1, SGPP1, SGPL1 and GAPDH were described previously [6, 33]. To limit the analysis of the expression of chemokines to the epithelial cell subsets only, we normalized our data to the keratin 8 gene. Others were normalized to the expression level of the GAPDH gene.

### Thymic slice preparation and imaging by two-photon microcopy

Preparation of thymic slices and imaging using a two-photon laser microscope were performed as described with some modifications. We injected mice just prior to sacrifice with 100μg Dylight594 conjugated lectin to label the blood vessels. A thymic lobe was embedded on a hand­made plastic pedestal with low-melting agarose(Lanzo). The thymic slice was cut every 400 μm using a vibratome (VT1000S, LEICA), and incubated with complete medium (RPMI-1640 containing 10% FCS, 50 μM 2-mercaptoethanol, 10 mM HEPES and antibiotics) for a few minutes at room temperature. The slices were placed onto a Millicell insert (30-mm organotypic PTFE; Millipore) in a 3.5cm plastic dish filled with 1 ml complete medium and then enclosed using silicone grease. The sorted CD4SP thymocytes from wild type B6 mice were labelled with 1 μM CFSE (Invitrogen) in 1×10^7^/ml of RPMI-1640 containing 1% FCS for 10 min at 37°C. The 4×10^5^ labelled CD4SP thymocytes were suspended in 20μl complete medium and loaded onto slices from Aire^−/−^ or Aire^+/+^ mice, and the cells on the slice were incubated for 3h at 37°C/5% CO2 to allow cells to enter the tissue. For the chemokine rescue assay, the CD4SP cells were incubated with with 200ng/ml CCL19 for 2 minutes and then loaded on the thymic tissues. Two-photon laser scanning microscopy was performed with an upright TCS SP5 microscope equipped with a 20× 1.0 NA water immersion objective (Leica) and a Chamelion VISION2 laser (COHERNT). For two-photon excitation, the Chamelion VISION2 laser was tuned to 858 nm. Dylight594 and CFSE emission was detected with nondescanned detectors fitted with 575-630nm and 495-540nm. To generate time-lapse series, stacks of 13-17 x-y sections with 2.5μm spacing were acquired every 20 s with 1.7× electronic zoom providing imaging volumes 32-42μm in depth and 151-153 μm in width. Imaging was performed 50-80μm beneath the cut surface of the slice. Image stack sequences were transformed into volume-rendered four-dimensional movies. From the x, y, z coordinates of cell centroids, cellular motility parameters were calculated as described [34].

### Data analysis

Volocity (PerkinElmer) was used for three-dimensional image analysis and automated tracking of cells. The accuracy of the automated tracking was manually controlled, and only tracks with durations of > 60 s were included in the analysis. Average cell velocity and dispalcement were calculated using Volocity. Statistical analysis was performed with GraphPad Prism 6. Results are displayed as individual data points plus median or mean ± SEM summarizing collective data from all experiments performed. All significant values were determined using the unpaired two-tailed t test or calculated by ANOVA tests. Throughout the text, figures, and figure legends, the following terminology is used to denote statistical significance: *p < 0.05, **p < 0.01. ***p < 0.001. *NS*, no significance.

## SUPPLEMENTARY MATERIAL















## Abbreviations

Aire: Autoimmune regulator
mTECs: medullary thymic epithelial cells
TRA: tissue-restricted antigen
S1P1: sphingosine-1-phosphate receptor 1
CMJ: cortical-medullary junction
KLF: Kruppel-like transcription factor
