# Supplementary material for: Critical role of SP thymocyte motility in regulation of thymic output in neonatal Aire−/− mice
Source: Oncotarget. 2016 Dec 11;8(1):83–94. doi: 10.18632/oncotarget.13909 (PMC5352200; doi:10.18632/oncotarget.13909)
Supplement: Supplementary file 1 [file oncotarget-08-83-s001.pdf]

Critical role of SP thymocyte motility in regulation of thymic output in neonatal *Aire*$^{-/-}$ mice

Supplementary Material

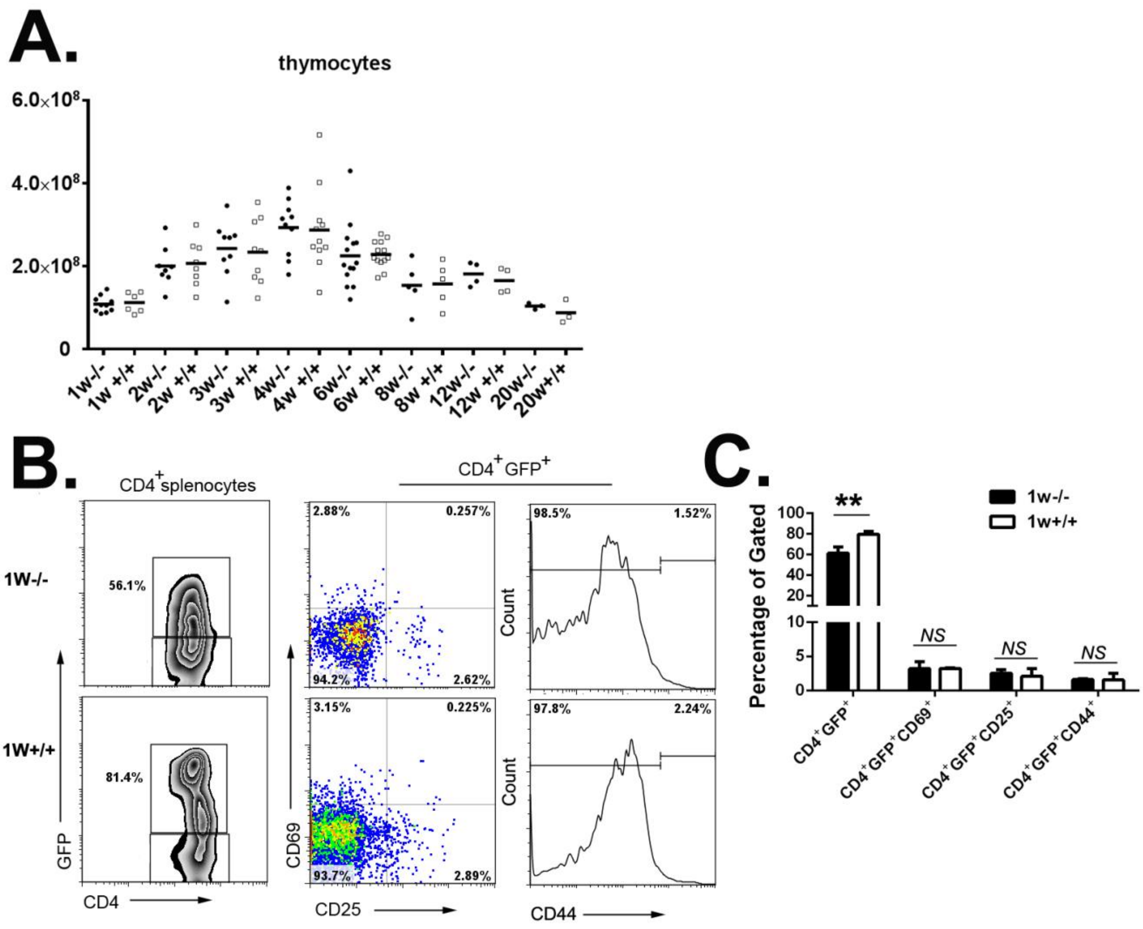

Supplementary Figure S1

Total number of thymocytes and RTE phenotype. A. The cell number of thymocytes from 1-20 weeks old Aire KO mice and littermates. **B.** Representative dot plots of GFP expression on CD4^{+} splenocytes from 1-week old *Aire*$^{-/-}$ RAG-GFP mice and littermates were shown on the left panel. CD69,CD25 and CD44 expression on CD4^{+}GFP^{+} RTEs were shown on the right panel. **C.** The ratio of RTEs and CD69,CD25 and CD44 expression on CD4^{+}GFP^{+} RTEs were summarized. Data from 5 mice are shown as mean ± SD. ** *p*<0.01, *** *p*<0.001. *NS*, no significance.

Supplementary Movies

1. Supplementary Movie 1 (1.14MB)

   Migration of CD4SP cells(green) within the medulla of 2 weeks-old *Aire*$^{-/-}$ mice. Blood vessels(red).

2. Supplementary Movie 2 (1.14MB)

   Migration of CD4SP cells(green) within the medulla of 2 weeks-old *Aire*$^{+/+}$ mice. Blood vessels(red).

3. Supplementary Movie 3 (1.15MB)

   Migration of CD4SP cells(green) within the medulla of 6 weeks-old *Aire*$^{-/-}$ mice. Blood vessels(red).

4. Supplementary Movie 4 (1.16MB)

   Migration of CD4SP cells(green) within the medulla of 6 weeks-old *Aire*$^{+/+}$ mice. Blood vessels(red).

5. Supplementary Movie 5 (1.16MB)

   Migration of CD4SP cells(green) incubated with CCL19 within the medulla of 2 weeks-old *Aire*$^{-/-}$ mice. Blood vessels(red).

6. Supplementary Movie 6 (1.21MB)

   Migration of CD4SP cells(green) incubated with CCL19 within the medulla of 2 weeks-old *Aire*$^{+/+}$ mice. Blood vessels(red).

For Supplementary Videos 1–6 see Supplementary Files.
